# Bromo-Cyclobutenaminones as New Covalent UDP-*N*-Acetylglucosamine Enolpyruvyl Transferase (MurA) Inhibitors

**DOI:** 10.3390/ph13110362

**Published:** 2020-11-03

**Authors:** David J. Hamilton, Péter Ábrányi-Balogh, Aaron Keeley, László Petri, Martina Hrast, Tímea Imre, Maikel Wijtmans, Stanislav Gobec, Iwan J. P. de Esch, György Miklós Keserű

**Affiliations:** 1 Division of Medicinal Chemistry, Amsterdam Institute of Molecular and Life Sciences (AIMMS), Faculty of Science, Vrije Universiteit Amsterdam, De Boelelaan 1108, 1081 HZ Amsterdam, The Netherlands; d.j.hamilton@vu.nl (D.J.H.); m.wijtmans@vu.nl (M.W.); i.de.esch@vu.nl (I.J.P.d.E.); 2 Medicinal Chemistry Research Group, Research Centre for Natural Sciences, Magyar tudósok krt 2, H-1117 Budapest, Hungary; abranyi-balogh.peter@ttk.hu (P.Á.-B.); aaron.keeley@ttk.hu (A.K.); petri.laszlo@ttk.hu (L.P.); 3 Faculty of Pharmacy, University of Ljubljana, Aškerčeva 7, SI-1000 Ljubljana, Slovenia; martina.hrast@ffa.uni-lj.si (M.H.); Stanislav.Gobec@ffa.uni-lj.si (S.G.); 4 MS Metabolomics Research Group, Research Centre for Natural Sciences, Magyar tudósok krt 2, H-1117 Budapest, Hungary; imre.timea@ttk.hu

**Keywords:** covalent inhibitor, MurA, cyclobutenaminone, antibacterial, irreversible

## Abstract

Drug discovery programs against the antibacterial target UDP-*N*-acetylglucosamine enolpyruvyl transferase (MurA) have already resulted in covalent inhibitors having small three- and five-membered heterocyclic rings. In the current study, the reactivity of four-membered rings was carefully modulated to obtain a novel family of covalent MurA inhibitors. Screening a small library of cyclobutenone derivatives led to the identification of bromo-cyclobutenaminones as new electrophilic warheads. The electrophilic reactivity and cysteine specificity have been determined in a glutathione (GSH) and an oligopeptide assay, respectively. Investigating the structure-activity relationship for MurA suggests a crucial role for the bromine atom in the ligand. In addition, MS/MS experiments have proven the covalent labelling of MurA at Cys115 and the observed loss of the bromine atom suggests a net nucleophilic substitution as the covalent reaction. This new set of compounds might be considered as a viable chemical starting point for the discovery of new MurA inhibitors.

## 1. Introduction

**Figure 1:**
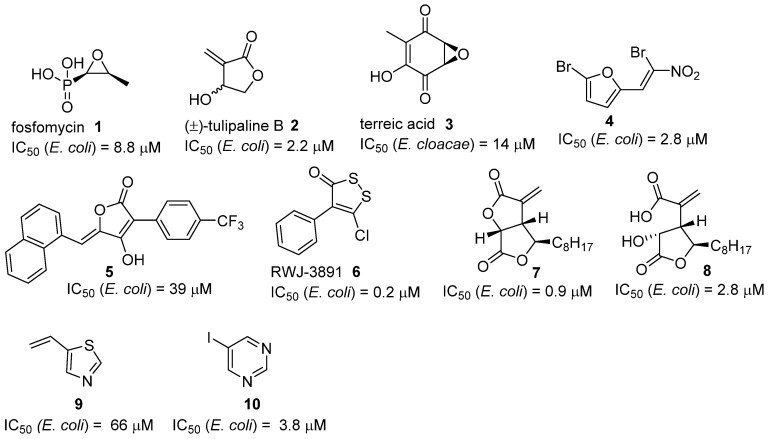
Known MurA inhibitors with small heterocyclic scaffolds.

MurA (UDP-*N*-acetylglucosamine enolpyruvyl transferase) is a key enzyme in the peptidoglycan biosynthesis that catalyzes the transfer of phosphoenolpyruvate (PEP) to UDP-*N*-acetylglucosamine (UNAG) [1]. Targeting the catalytic site of MurA leads to the inactivation of the enzyme that increases the osmotic vulnerability of bacteria [2]. MurA is a preferred antibacterial target, as there is no human orthologue for the enzyme. Known MurA inhibitors contain a three- (**1**,**3**), five-(**2**,**4**–**9**) or occasionally six-membered (**10**) heterocycle equipped with a halogen atom leaving group (**6**,**10**), or an epoxide ring (**1**,**3**) that are prone to nucleophilic substitution. Other inhibitors contain a double bond (**2**,**4**,**5**,**7**–**9**) that is available for Michael addition (Figure 1) [3,4,5,6].

Our attention was drawn to the cyclobutenone scaffold, as it also harbors a ring with electrophilic character. Cyclobutenones have received relatively little attention in the literature [7,8,9,10,11,12], and cyclobutyl compounds, in general, are underrepresented in most (fragment) screening libraries [13]. The ring strain of the cyclobutenone unit suggests a substantial reactivity as an electrophile [10,12,14]. Given the foreseen use as covalent fragments, the reactivity and stability in biological assays need to be balanced. Therefore, the electrophilic reactivity was carefully modulated by incorporating an amine functionality in the electrophilic core, giving cyclobutenaminones. As an additional advantage, appending the amine group to the core provides further chemical handles for growing any hit fragments. Last, cyclobutenaminones contain a double bond enabling the incorporation of, e.g., halogen atoms, that can target nucleophilic amino acid side chains, especially that of cysteines.

In continuation of our interests in finding new MurA inhibitors and in the use of the cyclobutyl motif in drug discovery [6,15,16,17], here we describe cyclobutenaminone derivatives with carefully-modulated electrophilic character as new warheads for covalently targeting the Cys115 residue in the active site of MurA.

## 2. Results and Discussion

**Scheme 1:**
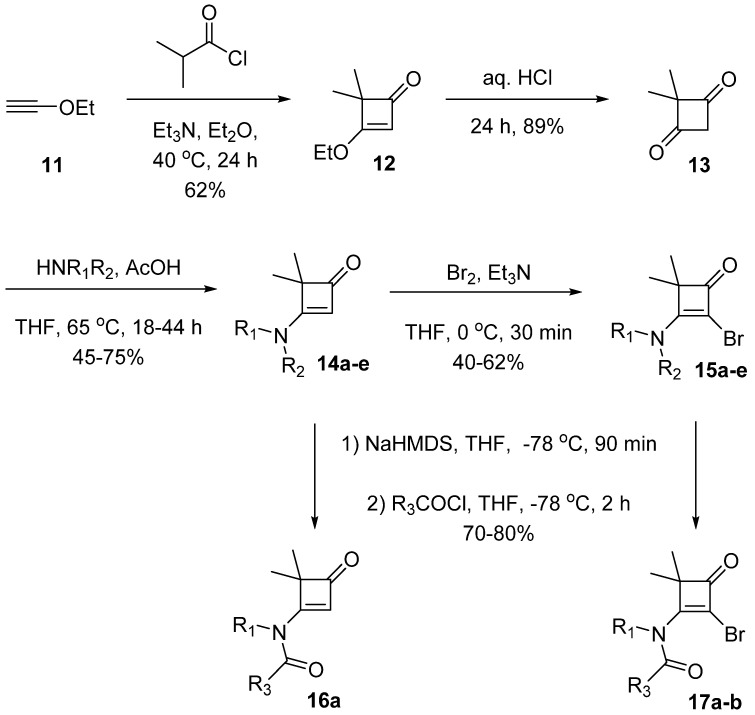
General synthesis route to various (bromo)enaminones and subsequent *N*-acylation of secondary enaminones. Table 1 shows the different substituents introduced (R_1,_ R_2_), while R_3_ = Me or Ph.

The synthesis of a small set of cyclobutenaminones was accomplished using a strategy based on that from Brand et al. (Scheme 1) [18]. The sequence began with ethoxyacetylene (**11**), which underwent a [2+2] cycloaddition [18,19,20,21] with the in situ generated ketene formed via the base-mediated HCl elimination from isobutyryl chloride. The two methyl groups were incorporated so as to restrict the nucleophilic character of enaminones (**14a**–**e**) to but one position in, e.g., the electrophilic bromination. The sequence furnished ethoxyenone (**12**) in moderate yield, which was transformed by acidic hydrolysis to 2,2-dimethylcyclobutane-1,3-dione (**13**) in high yield [18,19,20,21]. Next, dione **13** was condensed with various amines in the presence of AcOH as catalyst at 65 °C to generate the desired enaminones (**14a**–**e**) in moderate yields [18,22]. Bromination of all enaminones was achieved via electrophilic substitution using Br_2_ and base at 0 °C to produce bromoenaminones **15a**–**e** [18,22]. Selected compounds were subjected to *N*-acylation via deprotonation of the secondary enaminone in tetrahydrofurane (THF) at –78 °C by sodium bis(trimethylsilyl)amide (NaHDMS), followed by subsequent trapping by the relevant acid chloride. Conceivably, the acylation of **14a** to **16a** could also take place at the nucleophilic vinylic position. However, the correct regiochemistry was proven by 2D NMR experiments. The acylations resulted in the corresponding *N*-acyl-cyclobutenaminones (**16a** and **17a**–**b**) in good yields.

**Scheme 2:**
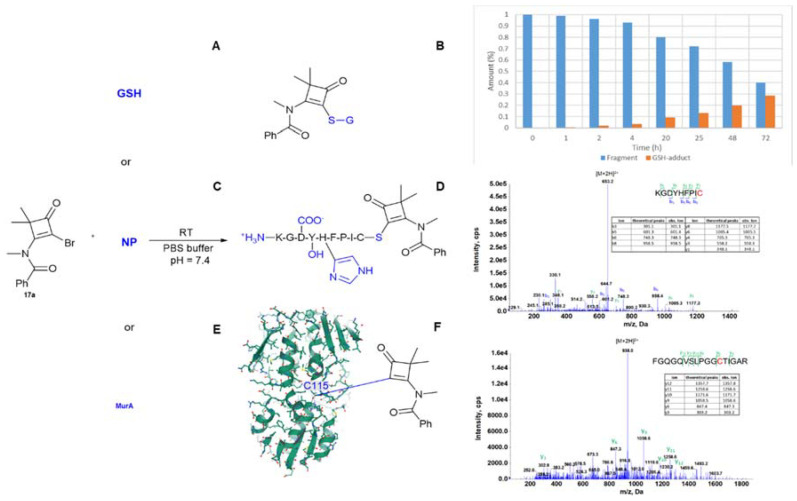
Labelling of (**A**) glutathione (GSH), (**C**) KGDHFPIC nonapeptide and (**E**) MurA with fragment **17a** together with (**B**) the measured consumption of the fragment (blue columns) and the increasing amount of the GSH-adduct (orange columns) in the GSH-assay, (**D**) the MS/MS spectrum of the Cys-labelled nonapeptide indicating the Cys-labelling together with the theoretical and observed ion peaks and (**F**) the MS/MS spectrum of the digested MurA fragment (amino acids 104–120) labelled on Cys115 together with the theoretical and observed ion peaks. For **E** the 1UAE X-ray structure has been used [27].

A library of thirteen fragments was prepared, containing nonbrominated (six) and brominated (seven) cyclobutenaminones, all of which are novel to the best of our knowledge (Table 1). This library was then screened against MurA from *E. coli* in order to identify possible starting points for the future development of covalent MurA inhibitors. The screening showed that the cyclobutenaminones with a vinylic proton (**14a**–**d**) do not give any substantial inhibition. Indeed, the amine substituent selected for balancing reactivity and stability (vide supra) will likely deactivate the double bond by its electron-donating character. We postulated that *N*-trifluoroethylation or *N*-acylation of the nitrogen atom might reactivate the system towards nucleophiles by withdrawing electron density from the conjugated system, but the results on **14e** and **16a** did not support this postulate. As an intermediate in the synthetic route, ethoxycyclobutenone (**12**) was also tested as the ethoxy unit could serve as an improved leaving group, but to no avail. Next, bromination of the vinylic position was explored for activation, bearing in mind that this modification has been successfully applied already in Diels-Alder reactions for improving reactivity [23]. Gratifyingly, several brominated cyclobutenaminones (**15d**, **17a**–**b**) inhibit MurA from *E. coli* at the 500 μM screening concentration (RA < 15%). Compounds containing amines alkylated with small substituents (**15a**–**c**) do not show any affinity to the protein, but the incorporation of the 4-methoxybenzyl group (**15d**) increases the affinity to IC_50_ = 363 μM. Turning attention to electron withdrawing groups once again, it was found that *N*-trifluoroethylation has no effect (**15e**), but the *N*-acetyl- and *N*-benzoyl-methylamino derivatives (**17a** and **17b**) substantially inhibit MurA activity. The time-dependent IC_50_ values of these compounds after 30 min are 138 μM and 128 μM, respectively—a substantial effect for such small fragments, with the latter possessing only thirteen heavy atoms. The time dependency of the IC_50_ values (see Supplementary Table S1) and the enhanced electrophilicity caused by the electron-withdrawing substituents suggest a covalent mechanism of action, which was confirmed by proving the labeling on Cys115 by MS/MS measurements for both compounds (Scheme 2E,F, Supplementary Figure S1). The MIC (Minimal Inhibitory Concentrations) values for the antimicrobial action of all compounds were determined against *S. aureus* (ATCC 29213) and *E. coli* (ATCC 25922) bacterial strains. These values were > 625 μM, implying that although MurA inhibition is clearly related to antibiotic action, more finetuning on these structures is needed on the path to a potential new class of antibiotics.

In order to characterize this new electrophilic chemotype, the cysteine reactivity of compound **17a** was evaluated in a GSH (glutathione)-based cysteine surrogate assay (Scheme 2A,B) [15]. The reaction of **17a** with GSH gives an adduct in the HPLC-MS-based assay (M + H^+^ = 535 Da], suggesting loss of the Br atom, and the conjugation reaction could be characterized with a rate constant of k_GSH_ = 0.0128 (M min)^−1^. Next, the selectivity of **17a** was explored using a nonapeptide assay (Scheme 2C,D) [15]. The KGDYHFPIC nonapeptide contains a cysteine but also other nucleophilic residues i.e., lysine, tyrosine, aspartate, proline, and histidine. As such, the nonapeptide can help to assess the selectivity between different biologically-relevant nucleophiles. In the case of **17a**, only the thiol group of the oligopeptide reacts with the warhead, indicating a high degree of cysteine specificity. To evaluate if the warhead is not too reactive for standard assay conditions, the aqueous stability of the compound (**17a**) was also investigated in PBS buffer (pH 7.4) [15]. The stability proves to be appropriate for biological investigations, as the t_1/2_ value for the aqueous degradation was determined to be 36.5 h at room temperature. The stability and bioavailability of these structures is also supported by the fact that interestingly, the rather unique bromocyclobutenaminone core has been incorporated both in the α4β1/α4β7 integrin antagonist prodrug, Zaurategrast [24,25], which progressed to phase II clinical trials [26], as well as in related compounds [22].

## 3. Materials and Methods

### 3.1. Synthesis and Characterisation of Compounds

All starting materials were obtained from commercial suppliers (primarily being Sigma-Aldrich (Swijndrecht, The Netherlands), Fluorochem (Hadfield, Derbyshire, UK) and CombiBlocks (San Diego, CA, USA)) and used without purification. Anhydrous Et_2_O, dichloromethane (DCM), acetonitrile (MeCN) and tetrahydrofurane (THF) were obtained by passing through an activated alumina column prior to use. All other solvents used were used as received unless otherwise stated. All reactions were carried out under a nitrogen atmosphere unless mentioned otherwise. TLC analyses were performed using Merck F_254_ (Merck KGaA, Darmstadt, Germany or VWR International B.V., Amsterdam, The Netherlands) aluminum-backed silica gel plates and visualized with 254 nm UV light or a potassium permanganate stain. Flash column chromatography was executed using Silicycle Siliaflash F_60_ silica gel (SiliCycle Inc., Quebec City, QC, Canada or Screening Devices, Amersfoort, The Netherlands) or by means of a Teledyne Isco CombiFlash (Teledyne Isco Inc., Lincoln, NE, USA or Beun de Ronde, Abcoude, The Netherlands) or a Biotage Isolera equipment using Biotage SNAP columns (Biotage AB, Uppsala, Sweden). All HRMS spectra were recorded on a Bruker micrOTOF mass spectrometer (Bruker Corp., Billerica, MA, USA) using ESI in positive-ion mode. All NMR spectra were recorded on either a Bruker Avance 300, Bruker Avance 500, or Bruker Avance 600 spectrometer (Bruker Corp., Billerica, MA, USA or Fällanden, Switzerland). The peak multiplicities are defined as follows: s, singlet; bs, broad singlet; d, doublet; t, triplet; q, quartet; p, pentet; dd, doublet of doublets; dt, doublet of triplets; td, a triplet of doublets; m, multiplet; app, apparent. The spectra were referenced to the internal solvent peak as follows: CDCl_3_ (^1^H = 7.26 ppm, ^13^C = 77.16 ppm), DMSO-d6 (^1^H = 2.50 ppm, ^13^C = 39.52 ppm). IUPAC names were adapted from ChemDraw Professional 16.0 (PerkinElmer). Purities were measured with the aid of analytical LC−MS using a Shimadzu LC-20AD liquid chromatography pump system (Shimadzu Corp., Kyoto, Japan or ‘s Hertogenbosch, The Netherlands) with a Shimadzu SPDM20A diode array detector (Shimadzu Corp., Kyoto, Japan) with the MS detection performed with a Shimadzu LC-MS-2010EV mass spectrometer (Shimadzu Corp., Kyoto, Japan) operating in both positive and negative ionization mode. The column used was an XBridge (C18) 5 μm column (50 mm × 4.6 mm) (Waters Corp., Milford, MA, USA or Phenomenex, Utrecht, The Netherlands). The following solutions are used for the eluents. Solvent A: H_2_O (+0.1% HCOOH) and solvent B: MeCN (+0.1% HCOOH). The eluent program used is as follows: flow rate: 1.0 mL/min, start 95% A in a linear gradient to 10% A over 4.5 min, hold 1.5 min at 10% A, in 0.5 min in a linear gradient to 95% A, hold 1.5 min at 95% A, total run time: 8.0 min. Compound purities were calculated as the percentage peak area of the analysed compound by UV detection at 254 nm.

### 3.2. GSH Reactivity and Aqueous Stability Assay

The assay was adapted from our former publication [15].

For the glutathione assay, 500 μM solution of the fragment (PBS buffer pH 7.4, 10% MeCN, 250 μL) with 200 μM solution of indoprofen (Merck KGaA, Darmstadt, Germany) as internal standard was added to 10 mM glutathione (Merck KGaA, Darmstadt, Germany) solution (dissolved in PBS buffer, 250 μL) in a 1:1 ratio. The final concentration was 250 μM fragment, 100 μM indoprofen, 5 mM glutathione and 5% MeCN (500 μL). The final mixture was analyzed by HPLC-MS (Shimadzu LCMS-2020) after 0, 1, 2, 4, 20, 25, 48, 72 h time intervals. Degradation kinetics were also investigated respectively using the previously described method, applying pure PBS buffer instead of the glutathione solution. In this experiment, the final concentration of the mixture was 250 μM fragment, 100 μM indoprofen and 5% MeCN. The AUC (area under the curve) values were determined via integration of HPLC spectra then corrected using the internal standard. The fragment AUC values were applied for ordinary least squares (OLS) linear regression and for computing the important parameters (kinetic rate constant, half-life time) a programmed excel (Visual Basic for Applications) was utilized. The data are expressed as means of duplicate determinations, and the standard deviations were within 10% of the given values.

The calculation of the kinetic rate constant for the degradation and corrected GSH-reactivity is as follows:

The reaction half-life for pseudo-first-order reactions is t_1/2_ = ln2/k, where k is the reaction rate. In the case of competing reactions (reaction with GSH and degradation), the effective rate for the consumption of the starting compound is k_eff_ = k_deg_ + k_GSH_. When measuring half-lives experimentally, the t_1/2(eff)_ = ln2/(k_eff_) = ln2/(k_deg_ + k_GSH_). In our case, the corrected k_deg_ and k_eff_ (regarding blank and GSH-containing samples, respectively) can be calculated by linear regression of the data points of the kinetic measurements. The corrected k_GSH_ is calculated by k_eff_ − k_deg_, and finally, the half-life time is determined using the equation t_1/2(GSH)_ = ln2/k_GSH_.

### 3.3. Oligopeptide Selectivity Assay

The assay was adapted from our former publication [15].

For the nonapeptide assay, a 2 mM solution of the fragment (PBS buffer pH 7.4 with 20% MeCN) was added to 200 μM nonapeptide solution (PBS buffer pH 7.4) in a 1:1 ratio. The final assay mixture contained 1 mM fragment, 100 μM peptide and 10% MeCN. Based on the GSH reactivity, the applied incubation time was 24 h.

### 3.4. LC-MS/MS Measurement and Data Analysis of the Nonapeptide Reactivity Assay

A Sciex 6500 QTRAP triple quadrupole—linear ion trap mass spectrometer, equipped with a Turbo V Source in electrospray mode (AB Sciex Pte. Ltd., Framingham, MA, USA) and an Agilent 1100 Binary Pump HPLC system (Agilent Technologies, Waldbronn, Germany) equipped with an autosampler was used for LC-MS/MS analysis. Data acquisition and processing were performed using Analyst software version 1.6.2 (AB Sciex Pte. Ltd., Framingham, MA, USA). Chromatographic separation was achieved by Purospher STAR RP-18 endcapped (50 mm × 2.1 mm, 3µm) LiChocart^®^ 55-2 HPLC Cartridge (Merck KGaA, Darmstadt, Germany). The sample was eluted with gradient elution using solvent A (0.1% HCOOH in water) and solvent B (0.1% HCOOH in MeCN). Flow rate was set to 0.5 mL/min. The initial condition was 5% B for 2 min, followed by a linear gradient to 95% B for 6 min, followed by holding at 95% B 6–8 min; and from 8 to 8.5 min back to the initial condition with 5% eluent B and held for 14.5 min. The column temperature was kept at room temperature and the injection volume was 10 µL. Nitrogen was used as the nebulizer gas (GS1), heater gas (GS2), and curtain gas with the optimum values set at 35, 45 and 45 (arbitrary units). The source temperature was 450 °C and the ion spray voltage set at 5000 V. The declustering potential value was set to 150 V. Information Dependent Acquisition (IDA) LC-MS/MS experiment was used to determine if the fragment binding was specific to thiol residues or not. An enhanced MS scan was applied as the survey scan and enhanced product ion (EPI) was the dependent scan. The collision energy in EPI experiments was set to 30 eV with a collision energy spread (CES) of 10 V. The identification of the binding position of the fragments to the nonapeptide was performed using GPMAW 4.2. software.

### 3.5. Tryptic Digestion of MurA

The tryptic digestion method was adapted from our former publication [15].

Briefly, 50 μL of MurA (42 μM) and 10 μL 0.2% (*w*/*v*) RapiGest SF (Waters Corp., Milford, MA, USA) solution buffered with 50 mM ammonium bicarbonate (NH_4_HCO_3_) were mixed (pH = 7.8). 4.5 μL of 45 mM DTT (~200 nmol) in 100 mM NH_4_HCO_3_ was added and the mixture kept at 37.5 °C for 30 min. After cooling the sample to room temperature, 7.5 μL of 100 mM iodoacetamide (750 nmol) in 100 mM NH_4_HCO_3_ was added and the mixture placed in the dark at room temperature for 30 min. The reduced and alkylated protein was then digested by 10 μL (1 mg mL^−1^) trypsin (the enzyme-to-protein ratio was 1:10) (Sigma, St Louis, MO, USA). The sample was incubated at 37 °C overnight. To degrade the surfactant, 7 μL of HCOOH (500 mM) solution was added to the digested HDAC8 sample to obtain the final 40 mM (pH ≈ 2) solution which was incubated at 37 °C for 45 min. For LC-MS analysis, the acid-treated sample was centrifuged for 5 min at 13,000 rpm.

### 3.6. LC-MS/MS Measurements on Digested MurA

A QTRAP 6500 triple quadruple—linear ion trap mass spectrometer, equipped with a Turbo V source in electrospray mode (AB Sciex Pte. Ltd., Framingham, MA, USA) and an Agilent 1100 Binary Pump HPLC system (Agilent Technologies, Waldbronn, Germany) equipped with an autosampler was used for LC-MS/MS analysis. Data acquisition and processing were performed using Analyst software version 1.6.2 (AB Sciex Pte. Ltd., Framingham, MA, USA). Chromatographic separation was achieved by using the Discovery^®^ BIO Wide Pore C-18-5 (250 mm × 2.1 mm, 5 μm). The sample was eluted with a gradient of solvent A (0.1% HCOOH in water) and solvent B (0.1% HCOOH in MeCN). The flow rate was set to 0.2 mL min^−1^. The initial conditions for separation were 5% B for 7 min, followed by a linear gradient to 90% B for 53 min, followed by 90% B for 3 min; over 2 min back to the initial conditions with 5% eluent B retained for 10 min. The injection volume was 10 μL (300 pmol on the column).

An Information-Dependent Acquisiton (IDA) LC-MS/MS experiment was used to identify the modified tryptic MurA peptide fragments. Enhanced MS scan (EMS) was applied as the survey scan and an enhanced product ion (EPI) was the dependent scan. The collision energy in EPI experiments was set to rolling collision energy mode, where the actual value was set on the basis of the mass and charge state of the selected ion. Further IDA criteria: ions greater than: 400.00 *m*/*z*, which exceeds 106 counts, exclude former target ions for 30 s after 2 occurrence(s). In EMS and in EPI mode, the scan rate was 1000 Da/s as well. Nitrogen was used as the nebulizer gas (GS1), heater gas (GS2), and curtain gas with the optimum values set at 50, 40 and 40 (arbitrary units). The source temperature was 350 °C and the ion spray voltage was set at 5000 V. The declustering potential value was set to 150 V. GPMAW 4.2. software was used to analyse a large number of MS-MS spectra and identify the modified tryptic MurA peptides.

### 3.7. MurA Assay

MurA_EC_ protein was recombinant, expressed in *E. coli.* [28] The inhibition of MurA was monitored with the colorimetric malachite green method in which orthophosphate generated during the reaction is measured. MurA enzyme (*E. coli*) was pre-incubated with the substrate UNAG and compound for 30 min at 37 °C. The reaction was started by the addition of the second substrate PEP, resulting in a mixture with a final volume of 50 μL. The mixtures contained: 50 mM Hepes, pH 7.8, 0.005% Triton X-114, 200 μM UNAG, 100 μM PEP, purified MurA (diluted in 50 mM Hepes, pH 7.8) and 500 μM of each tested compound dissolved in DMSO. All compounds were soluble in the assay mixtures containing 5% DMSO (*v*/*v*). After incubation for 15 min at 37 °C, the enzyme reaction was terminated by adding Biomol^®^ reagent (100μL) and the absorbance was measured at 650 nm after 5 min. All of the experiments were run in duplicate. Remaining activities (RAs) were calculated with respect to similar assays without the tested compounds and with 5% DMSO. The IC_50_ values, the concentration of the compound at which the remaining activity was 50%, were determined by measuring the remaining activities at seven different compound concentrations. The data are expressed as means of duplicate determinations, and the standard deviations were within 10% of the given values. A time-dependent inhibition assay was also performed. The IC_50_ values were determined at 0, 15 and 30 min of pre-incubation.

### 3.8. Antimicrobial Testing (MIC Determination)

Antimicrobial testing was carried out by the broth microdilution method in 96-well plate format following the CLSI guidelines and European Committee for Antimicrobial Susceptibility Testing recommendations. Bacterial suspension of specific bacterial strain, equivalent to 0.5 McFarland turbidity standard, was diluted with cation-adjusted Mueller Hinton broth to obtain a final inoculum of 10^5^ CFU/mL. Compounds dissolved in DMSO and inoculum were mixed together and incubated for 20–24 h at 37 °C. After incubation the minimal inhibitory concentration (MIC) values were determined by visual inspection as the lowest dilution of compounds showing no turbidity. The MICs were determined against *S. aureus* (ATCC 29213) and *E. coli* (ATCC 25922) bacterial strains. Tetracycline was used as a positive control on every assay plate, showing a MIC of 0.5 µg/mL and 1 µg/mL for *S. aureus* and *E. coli*, respectively.

### 3.9. Chemical Syntheses

3-Ethoxy-4,4-dimethylcyclobut-2-en-1-one (**12**)

To a stirred solution of isobutyryl chloride (10.7 mL, 102 mmol) and ethoxyacetylene **11** (50.0 mL, 205 mmol, 40% wt in hexanes) in Et_2_O (128 mL), Et_3_N (21.4 mL, 154 mmol) was added slowly over 5 min. The mixture was stirred at rt for 30 min before being heated at 40 °C for 24 h. The mixture was then allowed to cool. The precipitate was filtered and the filtrate was concentrated in vacuo. The crude product was purified over silica gel with a gradient of 10–40% EtOAc/cHex to afford the title compound **2** (8.90 g, 62% yield) as a yellow oil.

^1^H NMR (500 MHz, Chloroform-*d*) δ 4.78 (s, 1H), 4.21 (q, *J* = 7.1 Hz, 2H), 1.45 (t, *J* = 7.1 Hz, 3H), 1.24 (s, 6H). ^13^C NMR (126 MHz, Chloroform-*d*) δ 194.1, 190.4, 102.4, 69.4, 60.1, 19.7, 14.3. LC-MS: RT = 3.33 min, 99+% (254 nm), *m*/*z* [M + H]^+^ = 141. HRMS calculated for C_8_H_13_O_2_^+^ [M + H]^+^ = 141.0910, found 141.0923.

2,2-Dimethylcyclobutane-1,3-dione (**13**)

To a flask containing enol ether **12** (8.40 g, 59.9 mmol) was added HCl (2.0 M in H_2_O, 45.0 mL, 90.0 mmol) in one portion and the mixture was stirred vigorously at rt for 24 h. The product was extracted with DCM (3×). The organic layers were combined, dried over MgSO_4_, and concentrated in vacuo to afford the title compound **3** (6.20 g, 92% yield) as a flaky brown solid.

^1^H NMR (600 MHz, Chloroform-*d*) δ 3.92 (s, 2H), 1.28 (s, 6H). ^13^C NMR (151 MHz, Chloroform-*d*) δ 207.0, 73.0, 60.4, 17.6. LC-MS: RT = 1.78 min, 98% (254 nm), *m*/*z* [M + H]^+^ = 113. HRMS calculated for C_6_H_9_O_2_^+^ [M + H]^+^ = 113.0597, found 113.0603.

### 3.10. General Procedure A: Enaminone Formation

To a solution of dione **13** (1.0 eq) in THF (0.50 M) was added amine (1.1 eq), AcOH (1.1 eq) and a spatula of Na_2_SO_4_. The reaction mixture was stirred at 65 °C for the indicated time. The reaction mixture was allowed to cool to rt. The solids were filtered and the filtrate concentrated in vacuo. The residue was taken up in EtOAc and washed with satd. aq. Na_2_CO_3_ and brine. The organic layer was dried over Na_2_SO_4_, filtered and concentrated *in vacuo*. The crude product was purified over silica gel using the indicated gradient of MeOH/DCM to afford the product enaminone.

3-(Methylamino)-4,4-dimethylcyclobut-2-en-1-one (**14a**)

This compound was prepared according to General Procedure **A** using dione **13** (388 mg, 3.46 mmol), MeNH_2_ (2.0 M in THF, 1.90 mL, 3.81 mmol) and a reaction time of 40 h. Purification over silica gel using a gradient of 0–10% MeOH/DCM afforded the title compound **14a** (350 mg, 81%) as a pale brown solid.

Rotamers are observed in ratio ca. 1.0:0.1 in Chloroform-*d*. Only peaks corresponding to the major rotamer are reported. ^1^H NMR (500 MHz, Chloroform-*d*) δ 5.63 (br s, 1H), 4.59 (s, 1H), 2.99 (d, *J* = 5.0 Hz, 3H), 1.24 (s, 6H). ^13^C NMR (126 MHz, Chloroform-*d*) δ 192.2, 178.8, 95.7, 58.5, 31.8, 20.3. LC-MS: RT = 3.20 min, 99+% (254 nm), *m*/*z* [M + H]^+^ = 126. HRMS calculated for C_7_H_12_NO^+^ [M + H]^+^ = 126.0913, found 126.0912.

3-(Dimethylamino)-4,4-dimethylcyclobut-2-en-1-one (**14b**)

This compound was prepared according to General Procedure **A** using dione **13** (200 mg, 1.78 mmol), Me_2_NH (2.0 M in THF, 0.20 mL, 1.96 mmol) and a reaction time of 40 h. Purification over silica gel using a gradient of 0–10% MeOH/DCM afforded the title compound **14b** (191 mg, 77% yield) as a brown crystalline solid.

Rotamers are observed in ratio 1.0:1.0 in Chloroform-*d*. All peaks for both rotamers are reported. ^1^H NMR (500 MHz, Chloroform-*d*) δ 4.53 (s, 1H), 3.07 (s, 3H), 2.99 (s, 3H), 1.32 (s, 6H). ^13^C NMR (126 MHz, Chloroform-*d*) δ 190.9, 178.4, 96.0, 58.2, 40.1, 39.4, 21.2. LC-MS: RT = 2.34 min, 99 + % (254 nm), *m*/*z* [M + H]^+^ = 140. HRMS calculated for C_8_H_14_NO^+^ [M + H]^+^ = 140.1064, found 140.1066.

3-(Diethylamino)-4,4-dimethylcyclobut-2-en-1-one (**14c**)

This compound was prepared according to General Procedure **A** using dione **13** (200 mg, 1.78 mmol), Et_2_NH (0.20 mL, 1.96 mmol) and a reaction time of 40 h, followed by an additional portion of Et_2_NH (0.10 mL, 0.89 mmol) and stirring for a further 5 h. Purification over silica gel using a gradient of 0–10% MeOH/DCM afforded the title compound **14c** (175 mg, 59% yield) as a brown oil.

Rotamers are observed in ratio 1.0:1.0 in Chloroform-*d*. All peaks for both rotamers are reported. ^1^H NMR (500 MHz, Chloroform-*d*) δ 4.54 (s, 1H), 3.34 (q, *J* = 7.2 Hz, 2H), 3.27 (q, *J* = 7.2 Hz, 2H), 1.33 (s, 6H), 1.25 (t, *J* = 7.2 Hz, 3H), 1.22 (t, *J* = 7.2 Hz, 3H). ^13^C NMR (126 MHz, Chloroform-*d*) δ 190.9, 177.5, 95.6, 58.4, 44.6, 44.1, 21.4, 14.2, 12.3. LC-MS: RT = 3.06 min, 99+% (254 nm), *m*/*z* [M+H]^+^ = 168. HRMS calculated for C_10_H_18_NO^+^ [M + H]^+^ = 168.1377, found 168.1382.

3-((4-Methoxybenzyl)(methyl)amino)-4,4-dimethylcyclobut-2-en-1-one (**14d**)

This compound was prepared according to General Procedure **A** using dione 13 (200 mg, 1.78 mmol), (4-methoxybenzyl)-*N*-methylamine (0.29 mL, 1.96 mmol) and a reaction time of 40 h. Purification over silica gel using a gradient of 0–10% MeOH/DCM afforded the title compound **14d** (330 mg, 75% yield) as a viscous brown oil.

Rotamers are observed in ratio 1.0:1.0 in Chloroform-*d*. All peaks for both rotamers are reported. ^1^H NMR (600 MHz, Chloroform-*d*) δ 7.18–7.12 (m, 2H), 6.93–6.88 (m, 2H), 4.70 (s, 1H), 4.59 (s, 1H), 4.42 (s, 2H), 4.29 (s, 2H), 3.82 (s, 3H), 3.81 (s, 3H), 2.95 (s, 3H), 2.80 (s, 3H), 1.38 (s, 6H), 1.34 (s, 6H). ^13^C NMR (151 MHz, Chloroform-*d*) δ 190.9, 190.8, 178.4, 178.3, 159.6, 129.1, 128.7, 126.9, 126.7, 114.5, 114.4, 96.2, 95.9, 58.4, 58.3, 56.2, 55.4, 55.4, 55.3, 36.6, 36.3, 21.4, 21.1. LC-MS: RT = 3.62 min, 99+% (254 nm), *m*/*z* [M + H]^+^ = 246. HRMS calculated for C_15_H_20_NO_2_^+^ [M + H]^+^ = 246.1489, found 246.1489.

4,4-Dimethyl-3-(methyl(2,2,2-trifluoroethyl)amino)cyclobut-2-en-1-one (**14e**)

This compound was prepared according to General Procedure **A** using dione **13** (140 mg, 1.25 mmol), (2,2,2-trifluoroethyl)-methylamine (0.14 mL, 1.37 mmol) and a reaction time of 16 h. Purification over silica gel using a gradient of 0–10% MeOH/DCM afforded the title compound **14e** (197 mg, 76% yield) as a brown oil.

Rotamers are observed in ratio 1.0:0.8 in Chloroform-*d*. All peaks for both rotamers are reported. ^1^H NMR (500 MHz, Chloroform-*d*) δ 4.67 (s, 1H), δ 4.70 (s, 1H), 3.80 (q, *J* = 8.6 Hz, 2H), 3.75 (q, *J* = 9.0 Hz, 2H), 3.20 (s, 3H), 3.09–3.10 (m, 3H), 1.35 (s, 6H), 1.32 (s, 6H).^13^C NMR (151 MHz, Chloroform-*d*) δ 190.63, 179.51, 179.47, 124.47 (q, *J* = 281.9 Hz), 123.70 (q, *J* = 281.9 Hz), 99.72, 99.03, 54.29 (q, *J* = 34.1 Hz), 53.51 (q, *J* = 34.1 Hz), 39.06, 21.19, 21.08. LC-MS: RT = 3.30 min, 99+% (254 nm), *m*/*z* [M + H]^+^ = 208. HRMS calculated for C_9_H_13_F_3_NO^+^ [M + H]^+^ = 208.0944, found 208.0947.

### 3.11. General Procedure B: Bromination

A solution of enaminone (1.0 eq) and Et_3_N (2.0 eq) in THF (0.10 M) at 0 °C was treated dropwise with a solution of Br_2_ (1.1 eq) in THF (2.0 mL). The reaction mixture was stirred at 0 °C for the indicated time. The mixture was diluted with EtOAc and washed with satd. aq. NaHCO_3_ and brine. The organic layer was dried over MgSO_4_, filtered and concentrated *in vacuo*. The crude product was purified over silica gel using the indicated gradient of MeOH/EtOAc followed by reversed-phase chromatography on C18 silica gel using the indicated gradient of MeCN/H_2_O to afford the product bromoenaminone.

2-Bromo-4,4-dimethyl-3-(methylamino)cyclobut-2-en-1-one (**15a**)

This compound was prepared according to General Procedure B using enaminone **14a** (80 mg, 0.64 mmol) and a reaction time of 1 h. Purification over silica gel using a gradient of 0–10% MeOH/EtOAc and over reversed-phase C18 silica gel using a gradient of 0–100% MeCN/H_2_O (+0.1% HCOOH) afforded the title compound **15a** (81 mg, 62% yield) as a pale yellow solid.

Rotamers are observed in ratio 1.0:0.6 in Chloroform-*d*. All peaks for both rotamers are reported. ^1^H NMR (600 MHz, Chloroform-*d*) δ 5.97 (br s, 1H), 5.55 (br s, 1H), 3.30 (d, *J* = 5.2 Hz, 3H), 3.12 (d, *J* = 5.2 Hz, 3H), 1.38 (s, 6H), 1.24 (s, 6H). ^13^C NMR (151 MHz, Chloroform-*d*) δ 187.8, 185.9, 177.6, 175.5, 72.8, 70.5, 59.1, 58.7, 31.6, 31.4, 20.8, 19.9. LC-MS: RT = 2.80 min, 99+% (254 nm), *m*/*z* [M + H]^+^ = 204 (light isotope). HRMS calculated for C_7_H_11_NOBr^+^ [M + H]^+^ = 205.9998 (heavy isotope), found 205.9997.

2-Bromo-4,4-dimethyl-3-(methylamino)cyclobut-2-en-1-one (**15b**)

This compound was prepared according to General Procedure B using enaminone **14b** (80 mg, 0.58 mmol) and a reaction time of 1 h. Purification over silica gel using a gradient of 0–10% MeOH/EtOAc and over reversed-phase C18 silica gel using a gradient of 0–100% MeCN/H_2_O (+0.1% HCOOH) afforded the title compound **15b** (59 mg, 47% yield) as a pale yellow solid.

Rotamers are observed in ratio 1.0:1.0 in Chloroform-*d*. All peaks for both rotamers are reported. ^1^H NMR (600 MHz, Chloroform-*d*) δ 3.35 (s, 3H), 3.07 (s, 3H), 1.32 (s, 6H). ^13^C NMR (151 MHz, Chloroform-*d*) δ 186.5, 174.5, 70.3, 58.7, 40.7, 39.6, 20.9. LC-MS: RT = 3.06 min, 99+% (254 nm), *m*/*z* [M + H]^+^ = 218 (light isotope). HRMS calculated for C_8_H_13_NOBr^+^ [M + H]^+^ = 218.0175 (light isotope), found 218.0182.

2-Bromo-3-(diethylamino)-4,4-dimethylcyclobut-2-en-1-one (**15c**)

This compound was prepared according to General Procedure B using enaminone **14c** (80 mg, 0.48 mmol) and a reaction time of 1 h. Purification over silica gel using a gradient of 0–10% MeOH/EtOAc and over reversed-phase C18 silica gel using a gradient of 0–100% MeCN/H_2_O (+0.1% HCOOH) afforded the title compound **15c** (49 mg, 42% yield) as a colourless oil.

Rotamers are observed in ratio 1.0:1.0 in Chloroform-*d*. All peaks for both rotamers are reported. ^1^H NMR (600 MHz, Chloroform-*d*) δ 3.64 (q, *J* = 7.2 Hz, 1H), 3.35 (q, *J* = 7.2 Hz, 1H), 1.30 (t, *J* = 7.2 Hz, 3H), 1.28 (t, *J* = 7.2 Hz, 3H). ^13^C NMR (151 MHz, Chloroform-*d*) δ 186.6, 173.9, 69.7, 58.8, 45.7, 43.0, 21.1, 14.4, 14.2. LC-MS: RT = 3.78 min, 97% (254 nm), *m*/*z* [M + H]^+^ = 248 (heavy isotope). HRMS calculated for C_10_H_17_NOBr^+^ [M + H]^+^ = 246.0488 (light isotope), found 246.0488.

2-Bromo-3-((4-methoxybenzyl)(methyl)amino)-4,4-dimethylcyclobut-2-en-1-one (**15d**)

This compound was prepared according to General Procedure B using enaminone **14d** (100 mg, 0.41 mmol) and a reaction time of 2 h. Purification over silica gel using a gradient of 0–10% MeOH/EtOAc and over reversed-phase C18 silica gel using a gradient of 0–100% MeCN/H_2_O (+0.1% HCOOH) afforded the title compound **15d** (51 mg, 39% yield) as a pale yellow oil.

Rotamers are observed in ratio 1:0.6 in Chloroform-*d*. All peaks for both rotamers are reported. ^1^H NMR (600 MHz, Chloroform-*d*) δ 7.25–7.22 (m, 2H), 7.17–7.14 (m, 2H), 6.95–6.91 (m, 4H), 4.75 (s, 2H), 4.41 (s, 2H), 3.82 (s, 3H), 3.82 (s, 3H), 3.16 (s, 3H), 2.95 (s, 3H), 1.39 (s, 6H), 1.35 (s, 6H). ^13^C NMR (151 MHz, Chloroform-*d*) δ 186.71, 186.68, 174.72, 174.28, 159.90, 159.82, 129.52, 128.88, 127.15, 125.90, 114.70, 114.60, 70.51, 70.45, 58.96, 58.93, 56.64, 55.50, 55.47, 54.65, 37.43, 36.27, 21.23, 20.94. LC-MS: RT = 4.24 min, 99+% (254 nm), *m*/*z* [M + H]^+^ = 324 (light isotope). HRMS calculated for C_15_H_19_NO_2_Br^+^ [M + H]^+^ = 326.0573 (heavy isotope), found 326.0573.

2-Bromo-4,4-dimethyl-3-(methyl(2,2,2-trifluoroethyl)amino)cyclobut-2-en-1-one (**15e**)

This compound was prepared according to General Procedure B using enaminone **14e** (80 mg, 0.39 mmol) and a reaction time of 1 h. Purification over silica gel using a gradient of 0–10% MeOH/EtOAc and over reversed-phase C18 silica gel using a gradient of 0–100% MeCN/H_2_O (+0.1% HCOOH) afforded the title compound **15e** (32% yield) as a pale yellow solid.

Rotamers are observed in ratio 1:0.4 in Chloroform-*d*. All peaks for both rotamers are reported. ^1^H NMR (600 MHz, Chloroform-*d*) δ 4.27 (q, *J* = 8.3 Hz, 2H), 3.79 (q, *J* = 8.3 Hz, 2H), 3.46 (s, 3H), 3.22 (s, 3H), 1.37 (s, 6H), 1.34 (s, 6H). ^13^C NMR (151 MHz, Chloroform-*d*) δ 186.51, 175.87, 175.66, 123.95 (q, *J* = 282.1 Hz), 123.51 (q, *J* = 282.1 Hz), 74.86, 74.20, 59.70, 59.61, 54.57 (q, *J* = 34.0 Hz), 52.41 (q, *J* = 34.0 Hz), 40.05, 39.13, 20.93, 20.84. LC-MS: RT = 3.86 min, 99+% (254 nm), *m*/*z* [M + H]^+^ = 286 (light isotope). HRMS calculated for C_9_H_12_NOF_2_Br^+^ [M + H]^+^ = 286.0049 (light isotope), found 286.0044.

### 3.12. General Procedure C: Enaminone N-Acylation

To a solution of enaminone (1.0 eq) in THF (0.025 M) at −78 °C was added NaN(SiMe_3_)_2_ (1.0 M in THF, 1.5 eq) dropwise. The mixture was stirred at this temperature for 90 min before dropwise addition of the acid chloride (1.2 eq). The reaction mixture was stirred for a further 2 h at −78 °C. Brine was added slowly at this temperature whilst stirring vigorously and the mixture was allowed to warm to rt. The volatiles were removed in vacuo and the residue was partitioned between EtOAc and water. The organic layer was washed with brine, dried over Na_2_SO_4_, and concentrated *in vacuo*. The crude product was purified over silica gel to afford the product *N*-acyl-enaminone.

*N*-(4,4-dimethyl-3-oxocyclobut-1-en-1-yl)-*N*-methylbenzamide (**16a**)

This compound was prepared according to General Procedure C using enaminone **14a** (80 mg, 0.64 mmol) and BzCl (0.09 mL, 0.77 mmol). Purification over silica gel using a gradient of 20–80% EtOAc/cHex afforded the title compound **16a** (72% yield) as a white solid.

No significant rotamers are observed in NMR spectra. ^1^H NMR (500 MHz, Chloroform-*d*) δ 7.56–7.52 (m, 3H), 7.49–7.45 (m, 2H), 4.83 (s, 1H), 3.35 (s, 3H), 1.41 (s, 6H). ^13^C NMR (151 MHz, Chloroform-*d*) δ 194.2, 175.9, 171.1, 134.1, 132.0, 129.0, 127.9, 111.8, 62.7, 36.7, 21.4. LC-MS: RT = 3.85 min, 99% (254 nm), *m*/*z* [M + H]^+^ = 230. HRMS calculated for C_14_H_16_NO_2_^+^ [M + H]^+^ = 230.1176 found 230.1171.

The regiochemistry of the acylation (i.e., acylation of N atom and not of vinyl position) was proven by 2D NMR (HSQC + HMBC)—the singlet signal counting for 1H has an associated ^13^C signal in HSQC and thus the vinyl position remains unsubstituted in the product.

*N*-(2-bromo-4,4-dimethyl-3-oxocyclobut-1-en-1-yl)-*N*-methylbenzamide (**17a**)

This compound was prepared according to General Procedure C using bromoenaminone **15a** (80 mg, 0.39 mmol) and BzCl (0.06 mL, 0.47 mmol). Purification over silica gel using a gradient of 20–70% EtOAc/cHex afforded the title compound **17a** (86 mg, 71% yield) as a white solid.

No significant rotamers are observed in NMR spectra. ^1^H NMR (600 MHz, Chloroform-*d*) δ 7.62–7.54 (m, 3H), 7.53–7.46 (m, 2H), 3.61 (s, 3H), 1.43 (s, 6H). ^13^C NMR (151 MHz, Chloroform-*d*) δ 191.4, 174.6, 170.1, 133.5, 132.5, 129.1, 128.5, 87.8, 63.5, 39.0, 21.2. LC-MS: RT = 4.55 min, 99+% (254 nm), *m*/*z* [M + H]^+^ = 308 (light isotope). HRMS calculated for C_14_H_15_BrNO_2_^+^ [M + H]^+^ = 310.0260 (heavy isotope), found 310.0254.

*N*-(2-bromo-4,4-dimethyl-3-oxocyclobut-1-en-1-yl)-*N*-methylacetamide (**17b**)

This compound was prepared according to General Procedure C using bromoenaminone **15a** (6 mg, 0.03 mmol) and AcCl (0.002 mL, 0.03 mmol). Extraction with DCM (5 mL) and water (2 × 1 mL) afforded the title compound **17b** (5.9 mg, 80% yield) as a white solid.

No significant rotamers are observed in NMR spectra. ^1^H NMR (500 MHz, DMSO-*d6*) δ 3.19 (s, 3H), 1.90 (s, 3H), 1.11 (s, 6H). ^13^C NMR (125 MHz, DMSO-*d6*) δ 186.3, 184.9, 175.3, 68.7, 40.9, 30.5, 20.8, 20.1. LC-MS: RT 1.53 min, 97% (254 nm), *m*/*z* [M + H]^+^ = 246 (heavy isotope). HRMS calculated for deacetylated C_7_H_11_BrNO^+^ [M-acetyl + H]^+^ = 204.0018 (light isotope), found 204.0018.

## 4. Conclusions

A new electrophilic warhead chemotype, the bromocyclobutenaminone scaffold, was designed as a thiol-labelling agent. It was shown that the incorporation of the bromine atom in the cyclobutenaminone core, sometimes in conjunction with an electron withdrawing group on the nitrogen atom, turns the inactive fragments into novel and useful covalent probes. The investigation of a set of compounds against MurA protein from *E. coli* led to the identification of fragments with moderate inhibitory activity. These compounds represent promising starting points for hit optimization studies and fragment growing might lead to new and potent MurA inhibitors.

## Figures and Tables

**Table 1:**
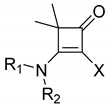
Structures and biological activity of synthesized compounds.

Compound	R_1_	R_2_	X	RA^a^ [%] at 500 µM and IC_50_ [µM]
**14a**	Me	H	H	93 ± 4
**14b**	Me	Me	H	99 ± 1
**14c**	Et	Et	H	96 ± 2
**14d**	Me	PMB ^b^	H	99 ± 3
**14e**	Me	F_3_CCH_2_	H	98 ± 7
**16a**	Me	PhCO	H	99 ± 8
**12**	-	-	-	98 ± 2
**15a**	Me	H	Br	100 ± 7
**15b**	Me	Me	Br	91 ± 5
**15c**	Et	Et	Br	88 ± 6
**15d**	Me	PMB^b^	Br	13 ± 1, IC_50_ = 363 ± 11
**15e**	Me	F_3_CCH_2_	Br	84 ± 4
**17a**	Me	PhCO	Br	12 ± 3, IC_50_ = 138 ± 9
**17b**	Me	MeCO	Br	8 ± 2, IC_50_ = 128 ± 10
**fosfomycin**	-	-	-	8.8 [3]

^a^ RA% refers to the remaining activity in the MurA (*E. coli*) biochemical assay with a fragment concentration of 500 μM with 30 min preincubation at 37 °C; ^b^ PMB: 4-methoxy-benzyl.

## Supplementary Materials

The following are available online at https://www.mdpi.com/1424-8247/13/11/362/s1, Table S1. Results of time-dependent IC_50_ measurements of **17b**; Figure S1. The MS/MS spectrum of **17b** modified MurA enzyme peptide [amino acids 104–120] together with the annotation of the peaks; NMR, LC-MS and HRMS data of key compound **17a**.
