# Supplementary material for: Bromo-Cyclobutenaminones as New Covalent UDP-N-Acetylglucosamine Enolpyruvyl Transferase (MurA) Inhibitors
Source: Pharmaceuticals (Basel). 2020 Nov 3;13(11):362. doi: 10.3390/ph13110362 (PMC7692735; doi:10.3390/ph13110362)

Supporting Information

Bromo-cyclobutenaminones as new covalent MurA inhibitors

David J. Hamilton[a,b], Péter Ábrányi-Balogh[b], Aaron Keeley[b], László Petri[b], Martina Hrast[c], Tímea Imre[d], Maikel Wijtmans[a], Stanislav Gobec[c], Iwan J. P. de Esch[a] ,and György Miklós Keserű[b,*]

[a] Division of Medicinal Chemistry, Amsterdam Institute of Molecular and Life Sciences (AIMMS), Faculty of Science, Vrije Universiteit Amsterdam, De Boelelaan 1108, 1081 HZ Amsterdam, The Netherlands.
[b] Medicinal Chemistry Research Group, Research Centre for Natural Sciences, Magyar tudósok krt 2, H-1117 Budapest, Hungary
[c] Faculty of Pharmacy, University of Ljubljana, Aškerčeva 7, SI-1000 Ljubljana, Slovenia
[d] MS Metabolomics Research Group, Research Centre for Natural Sciences, Magyar tudósok krt 2, H-1117 Budapest, Hungary
* corresponding author: keseru.gyorgy@ttk.mta.hu

Table of contents

Table S1. Results of time-dependent IC_{50} measurements of **17b** 2

Figure S1. The MS/MS spectrum of **17b** modified MurA enzyme peptide [104-120] together with the annotation of the peaks 2

NMR, LC-MS and HRMS data of key compound **17a** 3

Table S1. Results of time-dependent IC_{50} measurements of 17b

Preincubation time	IC_{50} (µM)
0 min	356 ± 29
15 min	149 ± 15
30 min	128 ± 10

Figure S1. The MS/MS spectrum of 17b modified MurA enzyme peptide [104-120] together with the annotation of the peaks

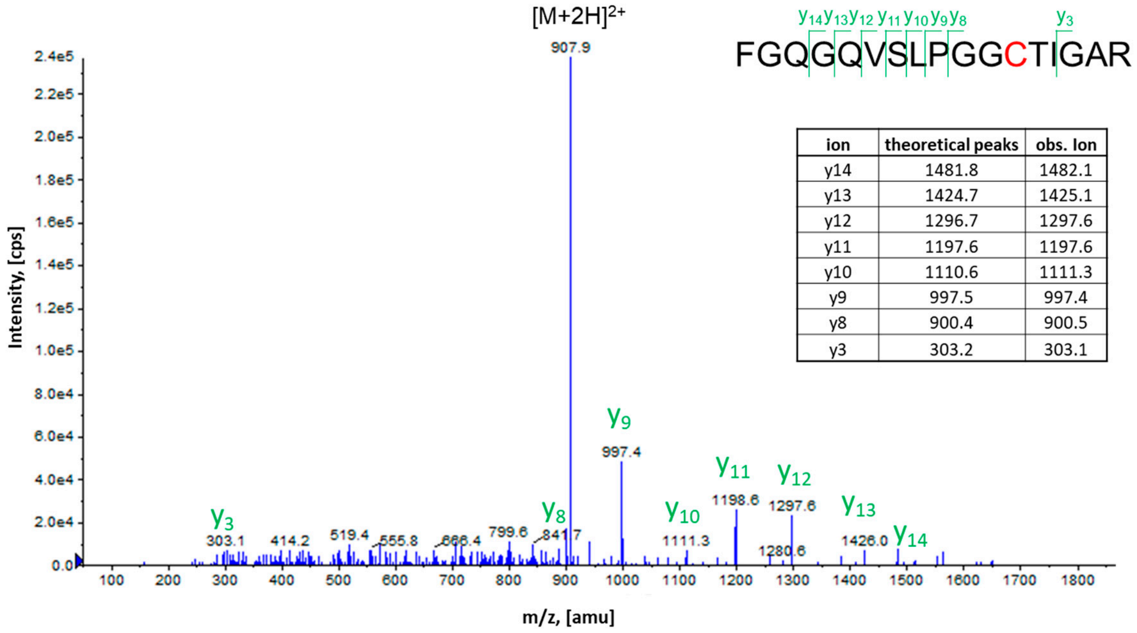

ion	theoretical peaks	obs. Ion
y14	1481.8	1482.1
y13	1424.7	1425.1
y12	1296.7	1297.6
y11	1197.6	1197.6
y10	1110.6	1111.3
y9	997.5	997.4
y8	900.4	900.5
y3	303.2	303.1

NMR, LC-MS and HRMS data of key compound 17a

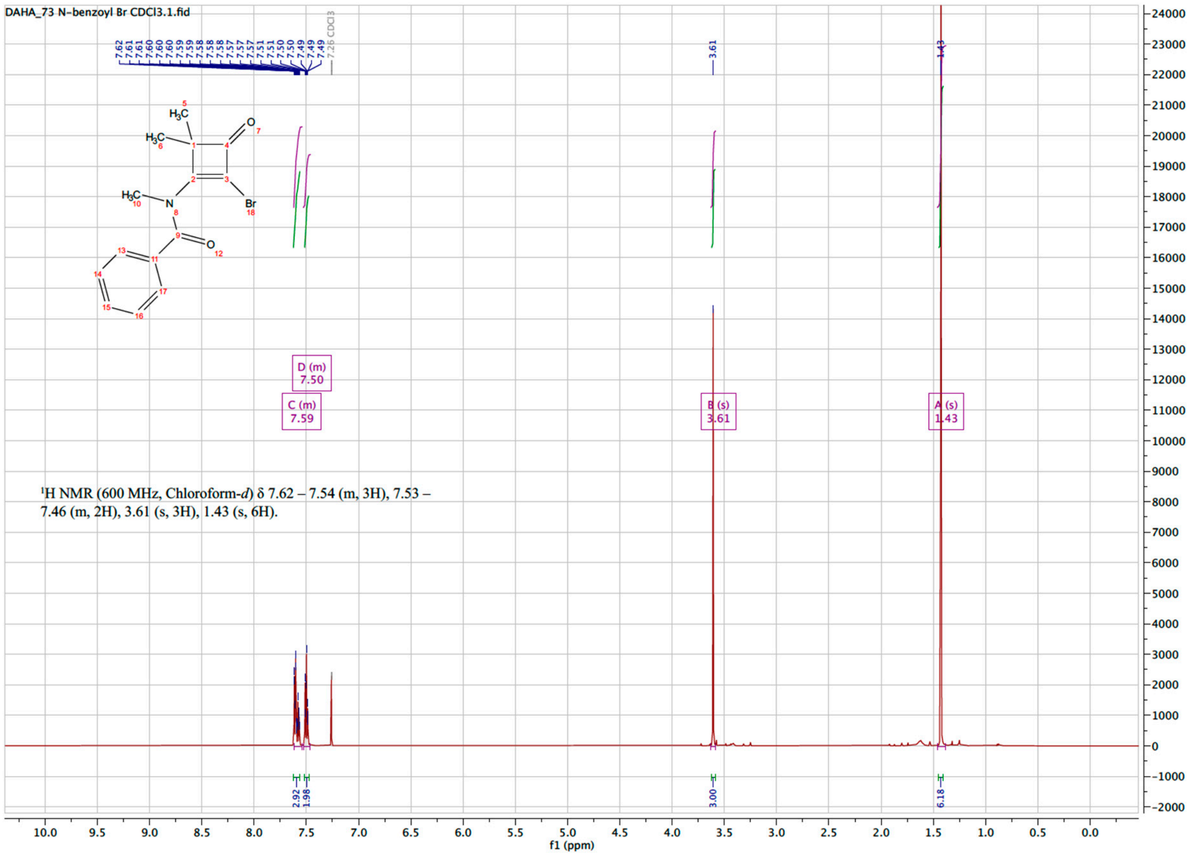

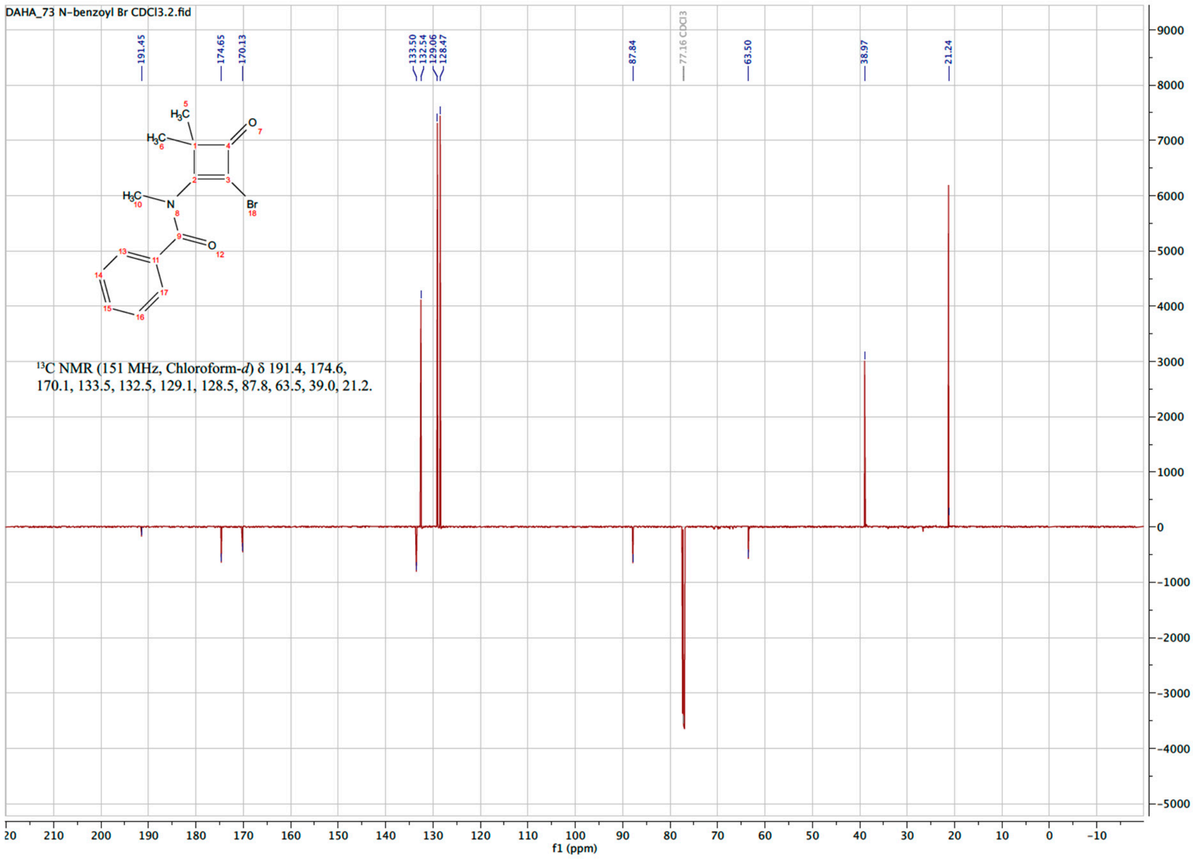

PDA Graph

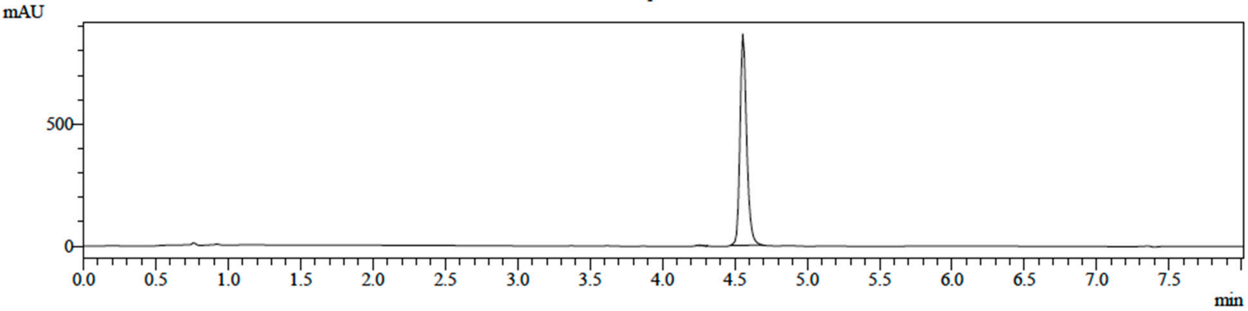

PeakTable

PDA Ch1 254nm 4nm

Peak#	Name	Ret. Time	Area	Area %
1		4.250	5177	0.188
2		4.551	2752417	99.812

MS Chromatogram

DAHA_73 N-benzoyl br C:\LabSolutions\Data\2019\2019-wk31-40\2019-wk31\DAHA_73 N-benzoyl br.lcd

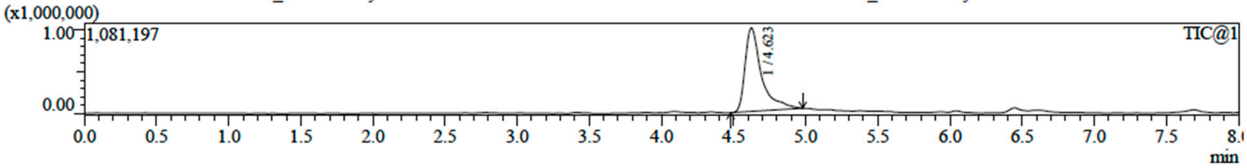

MS Spectrum Graph

#:1 Ret.Time:Averaged 4.610-4.630(Scan#:462-464)

BG Mode:Calc 4.480<->4.980(449<->499)

Mass Peaks:8 Base Peak:310.00(406224) Polarity:Pos Segment1 - Event1

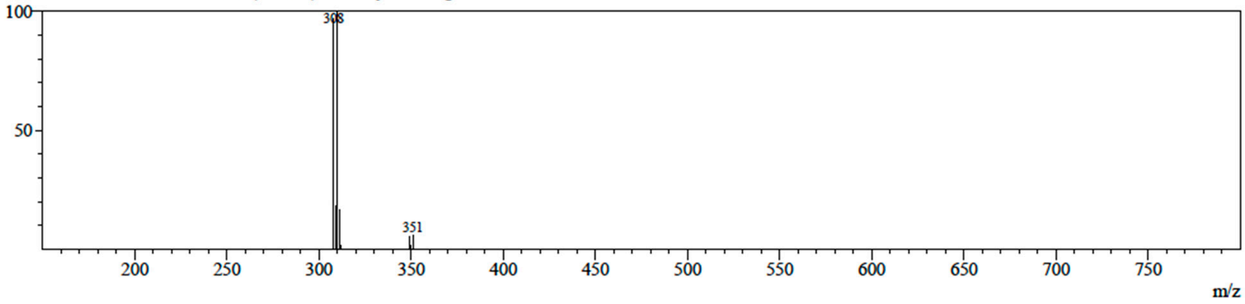

Supplement: Supplementary file 1 [file pharmaceuticals-13-00362-s001.pdf]
